# On ‘Flexible design in the stomatopod dactyl club’

**DOI:** 10.1107/S2052252523003408

**Published:** 2023-04-21

**Authors:** Stuart R. Stock

**Affiliations:** a Department of Cell and Developmental Biology and Simpson Querrey Institute, Northwestern University, Chicago, USA

**Keywords:** biomineralization, stomatopods, crystal orientation, composite materials

## Abstract

This commentary discusses loose versus tight control of biomineralization products and how this evolved flexibility. Concomitant improved functionality may be more widespread than commonly thought.

There are a surprisingly large number of biominerals, that is, minerals produced by living organisms, and these utilize a myriad of cations and anions, see Lowenstam & Weiner (1989 ▸). Researchers differentiate between biologically induced and biologically controlled biomineralization. In the former, the organism sets up conditions favorable for mineralization; induced mineralization can occur when normal tissue environments become deranged and leads, for example, to the formation of kidney stones or, following kidney transplants, to artery calcification. In biologically controlled biomineralization, the organism precisely controls mineral nucleation, growth and its cessation through space delineation, a preformed organic framework or matrix and a saturated solution or other precursors; thus, the same mineral product and morphologies occur within all individuals of a species. Examples include skeletal structural elements such as bones, teeth, external ‘armor’ and weapons, either for fighting or food gathering.

Mineralized tissues (*a*) can grow and be replaced over time, (*b*) can form and be added to over time [recording structures (Klevezal, 1996 ▸)] or (*c*) can be periodically shed and totally replaced. Considering the calcium phosphate and calcium carbonate mineralized tissues, examples of (*a*) include bone and its remodeling (Kenkre & Bassett, 2018 ▸), rodent incisors (Pugach & Gibson, 2014 ▸) and sea urchin teeth (Märkel *et al.*, 1977 ▸); of (*b*) fish otoliths (Campana & Neilson, 1985 ▸), tooth cementum (Naji *et al.*, 2022 ▸) and shark vertebrae (Natanson *et al.*, 2018 ▸); of (*c*) deer antlers (Kierdorf *et al.*, 2022 ▸) and stomatopod dactyl clubs (Christensen *et al.*, 2023 ▸). These three classes provide different windows into biomineralization and dactyl clubs, and their non-equilibrium mineral constituents are the topic discussed in a paper by Christensen *et al.* in this issue of **IUCrJ** .

Within the biomineralization community, there is a strong, largely unvoiced, presumption that biomineralization (except for pathologies such as ectopic mineral formation) is precisely controlled by the action of specific cells and macromolecules. Data strongly support this view in many cases, including the most heavily studied biomineralized tissues, bone or bone-analogs and tooth. The paper by Christensen *et al.* (2023 ▸) reports a case where portions of a mineralized tissue structure do not appear to be under tight control. In fact, Christensen *et al.* argue that relatively loose control of the mineral formed offers functional advantages to stomatopods.

The dactyl clubs of the stomatopod *Odontodactylus scylliarus* are an example of food-gathering weapons: the animal uses its pair of clubs ‘to destroy its prey with bullet-like acceleration (Christensen *et al.*, 2023 ▸).’ Previous research focused on the impact region which is certainly the place to start because the impact region experiences the strongest deformation. However, strong stress waves must propagate through the entire club, and the structure away from the impact zone, *i.e.* the sides of the clubs, is also essential to club functionality. These clubs are biocomposites of chitin and calcium carbonate and calcium phosphate minerals, and the microstructure and mineral crystallography of the side parts of the club are the focus of the paper by Christensen *et al.* (2023 ▸).

Stomatopod dactyl clubs are shed periodically, and Christensen *et al.* studied clubs taken from living animals and clubs at different times after shedding. Observing different time points allowed the investigators to understand changes in the clubs and to say something very interesting about structural variability in the sides of the clubs. Correlation of several advanced techniques (laboratory micro-computed tomography, synchrotron scanning microfluorescence and synchrotron X-ray microdiffraction mapping) revealed the 3D distribution of minerals, the cross-sectional distribution of Ca and the different crystallographic phases and their crystallographic texture (that is, the preferred orientations of the crystal axes relative to the anatomical directions). These investigators established most clubs’ sides crystallize to calcite and not to bioapatite, the dominant crystal type in the impact zone. In other clubs, substantial amorphous mineral remained. Further, crystallization can occur while the club is still functional, and the distribution of calcite crystallites can vary drastically from club to club.

Christensen *et al.* suggest that the variability in structure of the sides of clubs provides ‘design’ flexibility and provides a ‘good enough’ structure. The results also suggest further directions of research. For example, one wonders whether the banded structure in Fig. 6 reflects periodic changes in growth processes, like that in cementum (Naji *et al.*, 2022 ▸) or shark centra (Natanson *et al.*, 2018 ▸), or reflects structural modulation evolved to damp stress waves accompanying club strikes or to reflect stress waves back out to the target. Further, one wonders whether Sr and Zn content are also modulated in the banded zone: data suggest that Zn modulation marks changes in biomineralization, *e.g.* Stock *et al.* (2017 ▸) and Ryan *et al.* (2020 ▸).

The author agrees with Christensen *et al.* that their results suggest tightly controlled mineralization in the impact zone but loosely controlled mineralization in the sides of the clubs. This is an extremely important demonstration that mineralization within a single organ is spatially modulated with tightly controlled mineralization in one place and loosely controlled mineralization elsewhere. Similarly, the author suspects that intertubular and peritubular dentin form by tight and loose control, respectively (Stock *et al.*, 2014*a* ▸). It is interesting to consider whether spatial constraint might have a role in the loose versus tight control of biomineralization. Micropatterning experiments have shown, for example, that single-crystal calcite can be induced to grow in a very specific orientation through spatial constraint (Aizenberg *et al.*, 2003 ▸). Spatial constraint and limitations on ion transport may play a role in the transition of calcite composition in sea urchin teeth: from high Mg calcite in the first forming structures (plates and prisms) to very high Mg calcite in the later forming material which ‘glues’ the tooth together (Stock *et al.*, 2014*b* ▸). In fact, some of the diffraction patterns in Stock *et al.* (2014*b* ▸) appear to show more than two compositions of Mg-calcite are present, hinting at composition variation under loose control or at least under significant local perturbation.

One sometimes runs across the notion that the biomineralized structures we observe today evolved to be optimized structures, which is not a helpful viewpoint. Specific, highly functional features are likely to persist for long periods if they are good enough for their purpose, if other evolutionary changes do not incidentally alter them, or if the gap to superior structures is too wide for evolution to ‘jump’. In fact, this is the opinion offered by Christensen *et al.* at the end of their discussion, namely that the observed side-wall structure is ‘good enough’ for its purpose.
